# Genomic Characteristics of a Multidrug-Resistant Extraintestinal Pathogenic *Escherichia coli* RZ-13 Isolates from Diarrheic Calves with High Mortality in China

**DOI:** 10.3390/microorganisms14030521

**Published:** 2026-02-24

**Authors:** Di-Di Zhu, Liang Zhang, Shao-Hua Yang, Chuan-Hui Ge, Jia-Qi Chen, Teng-Fei Ma, Hong-Jun Yang

**Affiliations:** 1 Shandong Key Laboratory of Animal Disease Control and Breeding, Institute of Animal Science and Veterinary Medicine, Shandong Academy of Agricultural Sciences, Ji’nan 250100, China; 18831270392@163.com (D.-D.Z.);; 2 College of Veterinary Medicine, Shandong Agricultural University, Tai’an 271017, China

**Keywords:** extraintestinal pathogenic *Escherichia coli*, plasmid, antimicrobial resistance, whole genome sequencing

## Abstract

Extraintestinal pathogenic *Escherichia coli* (ExPEC) poses escalating threats to human and veterinary health amid rising antimicrobial resistance. We isolated a highly virulent ExPEC strain RZ-13 (ST345, O134:H21) from diarrheic calves at a large beef cattle farm in Rizhao City, and conducted whole genome sequencing, conjugation experiments, and antimicrobial susceptibility testing to elucidate its genomic architecture and resistance mechanisms. The RZ-13 genome comprises one chromosome and four plasmids. The chromosome harbors virulence factors for adhesion, invasion, biofilm formation, and iron acquisition. Notably, plasmids pRZ13-1 (265,777 bp, IncHI2-IncHI2A) and pRZ13-3 (74,304 bp, IncFII) carry the majority of resistance genes. Plasmid pRZ13-1 carries 25 resistance genes, including *bla_CTX-M-55_*, *floR*, *qnrS1*, *sul3*, and *tet(A)*, as well as a complete tellurite resistance gene cluster, *terABCDEFZY1*. Its multidrug resistance (MDR) region features an IS*26*-mediated tandem amplification and an approximately 29 kb inverted structure. Comparative analysis indicated that the MDR region carried by this plasmid is highly prevalent in both animal-derived and human-derived isolates. Plasmid pRZ13-3 harbors an IS*91*-mediated mobile region that integrates both antimicrobial resistance and stress adaptation genes, which have been repeatedly identified in plasmids from diverse sources, including animals and humans. Conjugation experiments confirmed both pRZ13-1 and pRZ13-3 plasmids are self-transmissible and confer multidrug-resistant phenotypes to recipient strains, with pRZ13-3 exhibiting an exceptionally high transfer frequency of 8.9 × 10^−2^, substantially exceeding that of previously reported IncFII plasmids. These findings demonstrate that pRZ13-1 and pRZ13-3 serve as critical vehicles for resistance dissemination through complex mobile genetic element structures and efficient horizontal transfer, highlighting the urgent need for surveillance of livestock-reservoir ExPEC to mitigate public health risks.

## 1. Introduction

The pervasive use of antimicrobial agents in both clinical medicine and food–animal production has markedly accelerated the emergence and dissemination of multidrug-resistant (MDR) bacteria across human, animal, and environmental reservoirs, thereby posing an escalating threat to global public health and ecological integrity [1]. *Escherichia coli* (*E. coli*), a versatile commensal and opportunistic pathogen, serves as a critical indicator organism for antimicrobial resistance surveillance due to its ubiquitous presence and remarkable genetic plasticity. In our previous investigation, we isolated a highly virulent MDR *E. coli* strain, designated RZ-13, from visceral specimens of neonatal calves exhibiting severe diarrhea. This strain harbors a diverse repertoire of resistance genes, including *bla_TEM-1_*, *tet(A)*, *sul3*, *rmtB*, and *qnrS1*, conferring resistance to β-lactams, tetracyclines, sulfonamides, aminoglycosides, and quinolones [2]. Notably, these genetic determinants were initially documented in human and animal isolates during the 1980s–2000s and have since become widely disseminated across porcine, avine, and bovine hosts, implicating cattle also as a significant reservoir for resistance gene amplification and transmission [3,4,5,6,7,8,9,10].

The emergence of MDR bacteria is fundamentally driven by selective pressure exerted by antimicrobial usage. In Europe, veterinary antimicrobial consumption surpassed human use by 2014, with extensive application of third- and fourth-generation cephalosporins correlating with elevated resistance rates in both animal-derived and human clinical *E. coli* isolates [11]. Similarly, tetracycline and polymyxin resistance in livestock-associated bacteria has been directly linked to corresponding veterinary antimicrobial administration [12,13,14]. Crucially, the persistence and dissemination of antimicrobial resistance genes (ARGs) are mediated by mobile genetic elements (MGEs), which facilitate horizontal gene transfer (HGT) even following bacterial cell death, thereby maintaining environmental reservoirs of resistance determinants [13]. Among MGEs, plasmids constitute the predominant vehicles for ARG dissemination, attributed to their autonomous replication capacity and interbacterial transferability. Comparative analyses of 8229 plasmid-borne ARGs demonstrated that approximately 87% of resistance genes are transferable between compatible plasmids, with incompatibility groups IncF (especially IncFII family plasmids) and IncHI2 representing particularly significant reservoirs [15]. IncFII plasmids frequently encode extended-spectrum β-lactamases, plasmid-mediated quinolone resistance determinants, and aminoglycoside-modifying enzymes, whereas IncHI2 “superplasmids” are distinguished by their extensive size and capacity to accumulate diverse resistance determinants, occasionally existing as multireplicon entities [16,17]. Complementing plasmid-mediated transfer, insertion sequences (ISs) and transposons critically modulate ARG acquisition and expression through promoter provision, hybrid promoter formation, and transposition-mediated genetic rearrangement [18,19].

Traditionally, antimicrobial resistance acquisition has been associated with fitness costs and attenuated virulence. However, contemporary high-risk clones, exemplified by carbapenem-resistant hypervirulent *Klebsiella pneumoniae* and *E. coli* sequence type 131 (ST131), have circumvented these constraints through MGE-driven accumulation of resistance and virulence determinants. The continuous acquisition of β-lactamase genes (*bla_CTX-M-15_*, *bla_CMY-2_*, *bla_NDM-1_*), fluoroquinolone resistance mutations, and intrinsic virulence factors—including siderophore systems, hemolysins, and adhesins—has generated synergistic combinations of MDR and hypervirulence, potentiated by antibiotic selection pressure [20,21].

This study focused on *E. coli* RZ-13, an MDR and highly pathogenic strain. We analyzed the genetic structure of its two plasmids (IncFII and IncHI/IncHI2), emphasizing resistance gene transferability to assess their potential role in disseminating antimicrobial resistance.

## 2. Materials and Methods

### 2.1. Isolation and Identification of E. coli RZ-13

The *E. coli* strain RZ-13 was isolated post-mortem from the spleen and lungs of neonatal calves that had succumbed to severe diarrhea and respiratory distress on a large-scale beef cattle farm in Rizhao City, Shandong Province, China. The outbreak persisted for approximately 50 days, during which 108 of 126 calves housed in the same barn became infected, resulting in 78 deaths. The infection and mortality rates were 85.71% and 72.22%, respectively. Clinically, affected calves primarily exhibited watery diarrhea and respiratory distress. The attending veterinarian sequentially administered commonly used antibiotics, including gentamicin, ampicillin, and fluoroquinolones, all of which proved ineffective. Subsequently, comprehensive control measures were implemented to interrupt pathogen transmission, including isolation of affected calves, separate feeding, replacement of breast milk with milk powder, and intensified disinfection. These interventions ultimately contained the outbreak [2]. Necropsy and bacteriological examination identified RZ-13 as the predominant pathogen in all deceased animals.

Previous studies have demonstrated that this *E. coli* strain RZ-13 is significantly more virulent than the reference strain CICC 24186 (an Enteroaggregative *E. coli* strain obtained from the China Center of Industrial Culture Collection) and induces severe organ damage in animal models [2]. Additionally, the strain showed resistance to multiple classes of antibiotics, including quinolones, sulfonamides, macrolides, tetracyclines, beta-lactams, and aminoglycosides [2]. However, our previous study only showed the pathogenicity of the strain was characterized solely through cell-adhesion assays and a murine infection model, leaving its complete genomic landscape unaddressed; herein, we employed PacBio platform sequencing to delineate the full genome and the genetic context of resistance determinants, and evaluated the transferability of these resistance genes by conjugation experiments.

### 2.2. Whole Genome Sequencing and Assembly of E. coli RZ-13

The RZ-13 strain stored at −80 °C was streaked onto LB agar plates and incubated at 37 °C overnight. A single colony was picked and inoculated into LB broth, then cultivated at 37 °C with shaking at 200 rpm until the culture reached the logarithmic phase. Genomic DNA was extracted and sent to Novogene Bioinformatics Technology Co., Ltd. (Beijing, China) for both Illumina PE150 and PacBio sequencing. The Illumina workflow has been described previously [2]. For PacBio sequencing, a SMRTbell™ Template Prep Kit 2.0 was used to construct SMRTbell libraries. Genomic DNA that passed electrophoretic quality control was sheared to the desired insert size using a Covaris g-TUBE. After DNA-damage repair and end-repair, hairpin adapters were ligated to both ends of the DNA fragments with DNA ligase, followed by purification with AMPure PB beads. Size-selected fragments were obtained using BluePippin, and the SMRTbell libraries were further enriched by AMPure PB beads. DNA-damage repair was performed again, followed by a final purification. Library concentration was determined with a Qubit fluorometer, and insert sizes were checked on an Agilent 2100 Bioanalyzer. Libraries were sequenced on the PacBio platform. Raw PacBio reads were assembled de novo with Canu v2.0 (https://github.com/marbl/canu/ (accessed on 22 September 2024)) to obtain an initial assembly representing the overall genomic structure [22]. This assembly was polished with Racon v1.4.13 using the PacBio reads for three iterative rounds, followed by three additional polishing rounds with Pilon v1.22 using Illumina PE150 (NovaSeq X Plus, Illumina, San Diego, CA, USA; v1.0 chemistry) reads [23,24]. The final assembly comprised one complete chromosome and four circularized plasmids.

### 2.3. Bioinformatic Analysis of E. coli RZ-13

Genome annotation of strain RZ-13 was initially performed using RAST (https://rast.nmpdr.org/ (accessed on 28 October 2024)) [25,26,27], followed by manual curation. Antimicrobial resistance genes (ARGs) and plasmid replicon types were identified using the CGE platform (https://www.genomicepidemiology.org/services/ (accessed on 10 December 2024)) [28], while virulence factors were detected through the Virulence Factor Database (VFDB) (http://www.mgc.ac.cn/cgi-bin/VFs/v5/main.cgi?func=VFanalyzer (accessed on 10 December 2024)) [29]. Circular plasmid maps were generated with CGView (https://stothardresearch.ca/cgview/, accessed on 24 April 2025) [30], and plasmid comparisons were visualized using Easyfig v2.2.5 [31]. BLASTN (https://blast.ncbi.nlm.nih.gov/Blast.cgi (accessed on 2 February 2025)) was used to align plasmids carrying resistance genes against the NCBI nucleotide database. Comparative plasmid visualization was further conducted using BRIG v0.95 [32].

### 2.4. Nucleotide Sequence Accession Numbers of E. coli RZ-13

The complete genome sequence of the *E. coli* RZ-13 strain was submitted to JBSTPK000000000.

### 2.5. Conjugation and Transformation Experiments of E. coli RZ-13

To evaluate the transferability of resistance genes harbored by strain RZ-13, conjugation experiments were performed. With *E. coli* RZ-13 as a donor strain and *E. coli* J53 as a recipient strain, both were cultured on BHA plates supplemented with appropriate concentrations of florfenicol and NaN_3_, then transferred to BHI broth and incubated at 37 °C, 200 rpm for 5–6 h. The bacterial cultures were adjusted to 0.5 McFarland turbidity, mixed at a 1:3 donor-to-recipient ratio, and mating was performed on BHA plates at 37  °C [33].

Transconjugants carrying pRZ13-1 were selected on BHA containing 8 μg/mL florfenicol and 64 μg/mL NaN_3_, while those carrying pRZ13-3 were selected on 128 μg/mL amikacin and 64 μg/mL NaN_3_. The recipient strain was selected using 64 μg/mL NaN_3_. Additionally, it has been demonstrated that the conjugative transfer ability of IncHI-type plasmids is temperature-sensitive, and its transfer efficiency is highest between 22 and 30 °C, while it is significantly inhibited at 37 °C. To assess the temperature sensitivity of pRZ13-1 transfer, additional conjugation assays were performed at 24 °C. The conjugation frequency equaled the number of transconjugants divided by the number of recipients. The successful transconjugants were further verified by PCR targeting *floR* and *rmtB* genes, respectively [34].

### 2.6. Antibiotic Susceptibility Testing

Four bacterial suspensions (RZ-13, J53-pRZ13-3, J53-pRZ13-1 and J53) adjusted to a McFarland turbidity of 0.5 were evenly spread onto agar plates for the antimicrobial assay, and CICC 24186 was used as the control. The antibiotics used for drug sensitivity testing were norfloxacin (10 μg), ofloxacin (5 μg), enrofloxacin (10 μg), sulfamethoxazole (23.75 μg Sulfonamide and 1.25 μg trimethoprim), chloramphenicol (30 μg), gentamicin (10 μg), doxycycline (30 μg), kanamycin (30 μg), ceftriaxone (30 μg), ceftazidime (30 μg), cefalexin (30 μg), cefoxitin (30 μg), ampicillin (10 μg), and amoxicillin (20 μg). The antibiotic susceptibility were determined and interpreted following the guidelines provided by the Clinical & Laboratory Standards Institute guidelines VET01S or the European Committee on Antimicrobial Susceptibility Testing [35,36,37,38,39,40].

## 3. Results and Discussion

### 3.1. The Genomic Profiles of E. coli RZ-13

PacBio sequencing generated 124,563 reads with an average length of 10,832 bp, providing approximately 280× genome coverage. Illumina PE150 sequencing produced 4,521,678 read pairs (150 bp each), resulting in an additional 280×coverage. The final assembly comprised one complete circular chromosome and four circularized plasmids. Subsequently, circularity was verified by the presence of overlapping ends in the PacBio assembly, manual inspection of the assembly graph, and the identification of characteristic replication-associated genes on each replicon. Lastly, by normalizing the average read depth of each plasmid to that of the chromosome, we estimated relative copy numbers. The coverage ratios for pRZ13-1, pRZ13-2, pRZ13-3, and pRZ13-4 were 1.2, 0.9, 3.4, and 1.1, respectively, suggesting that pRZ13-3 exists at a higher copy number compared to the other plasmids.

Whole genome sequencing (WGS) analysis results showed that strain RZ-13 was identified as ST345 by multilocus sequence typing (MLST) and had a serotype of O134:H21. This strain contains one chromosome and four plasmids (pRZ13-1, pRZ13-2, pRZ13-3, and pRZ13-4) (Table 1). The chromosome is 4,864,281 bp in length, carrying resistance genes *bla_TEM-1_* and *tet(A)*, and virulence factors related to adhesion, invasion, biofilm formation, regulatory factors, and metabolic regulation, which have been described in previous studies [2]. The plasmids pRZ13-1 and pRZ13-3 both carry numerous drug resistance genes and a few virulence factors. However, plasmids pRZ13-2 and pRZ13-4 do not carry any drug resistance genes or virulence factors (Table 1). The identified virulence factors provide plausible mechanisms for the observed clinical signs. Fimbrial genes (*fimH*, csgA) and biofilm-related genes *(csgABCDEFG*) promote colonization of the intestinal epithelium, contributing to diarrhea [41,42]. f Flagellar genes (*flgC*, *flgG*, *flgH*) facilitate bacterial motility and extraintestinal invasion, enabling dissemination to the lungs [43]. Type VI secretion system effectors (*hcpA*, *vgrG*) can induce local inflammatory responses, exacerbating tissue damage in both the gut and respiratory tract [44]. Thus, the combination of these non-classical factors can account for both diarrheic and respiratory symptoms in the absence of classical enterotoxins or pneumonia-associated toxins. Additionally, screening for classical fimbrial antigens associated with calf diarrhea revealed that the RZ-13 genome does not contain genes encoding F5 (K99), F41, or F17 fimbriae. However, the presence of type 1 fimbriae (*fimH*) and curli fibers (*csgA*) may compensate for the absence of these specific adhesins, as both are known to mediate adherence to host epithelial cells and contribute to intestinal colonization [42,43].

**Figure 1:**
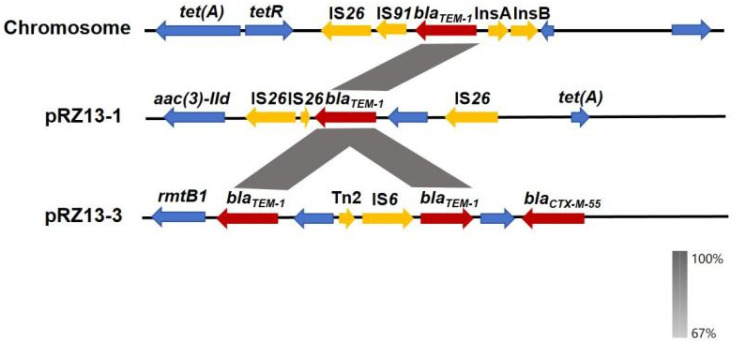
Genetic environment of the *bla_TEM-1_* gene commonly present in the chromosome of strain RZ-13 and the plasmids pRZ13-1 and pRZ13-3. The positions and transcriptional directions of the ORFs are denoted with arrows. Yellow indicates the transposon element, red indicates *bla_TEM-1_*, and blue represents the rest of the genetic environment.

Through a detailed analysis of the resistance genes carried by the strain RZ-13, an interesting phenomenon was discovered. The strain harbors a total of four *bla_TEM-1_* genes, located on the chromosome, plasmid pRZ13-1, and plasmid pRZ13-3 (two copies) (Figure 1). The nucleotide sequences and transcription directions of three of these copies are completely identical. However, the genetic backgrounds of the regions where these three *bla_TEM-1_* genes are located show significant differences, being associated with different types of transposons or insertion sequences. This suggests that the multi-site distribution of *bla_TEM-1_* may be caused by insertion rearrangement events mediated by different mobile genetic elements [45]. This structural diversity may play an important role in the stable maintenance and horizontal transfer of resistance genes [15]. The other copy located on pRZ13-3 is a truncated version, with a transcription direction opposite to the other three (Figure 1). This is likely the result of partial sequence deletion during transposition or recombination. The presence of multiple copies of the *bla_TEM-1_* gene may enhance the strain’s resistance to β-lactam antibiotics [46]. Moreover, the *bla_TEM-1_* genes located on plasmids are more likely to be horizontally transferred between bacteria, increasing the risk of *bla_TEM-1_* gene dissemination within bacterial populations.

### 3.2. Characteristics and Transferability of the Multidrug-Resistance Plasmid pRZ13-1

**Figure 2:**
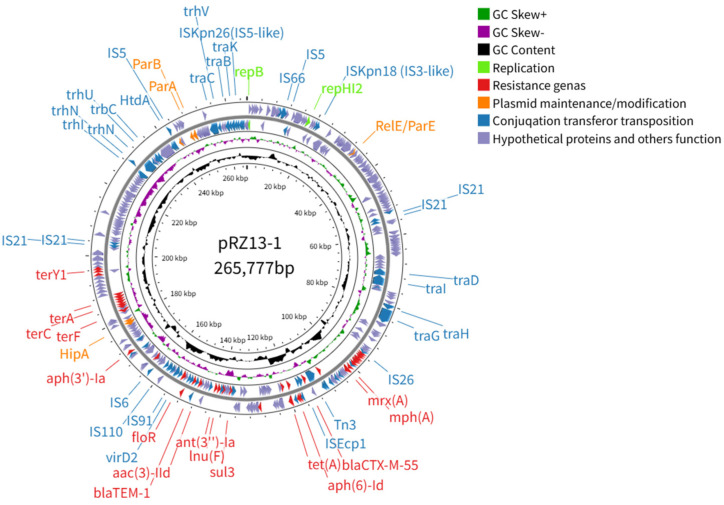
Circular map of plasmid pRZ13-1. Concentric rings (outermost to innermost) depict predicted coding sequences, GC skew, GC content, and scale in kilobases; ORFs are color-coded by functional category. The complete map was generated with CG View (https://stothardresearch.ca/cgview/ (accessed on 24 April 2025)).

Plasmid pRZ13-1 is a 265,777 bp IncHI2-IncHI2A hybrid replicon (46.80% G + C) encoding 356 open reading frames (ORFs) that include *aac(3)-IId*, *aph(6)-Id*, *aph(3″)-Ia/Ib*, *bla_CTX-M-55_*, *bla_TEM-1_*, *ant(3″)-Ia*, *mph(A)*, *mef(B)*, *mphR*, *lnu(F)*, *floR*, *qnrS1*, *sul3*, *tet(A)*, *dfrA14*, *cmlA*, *arr-2*, *mrx(A)*, *tet(R)* and *tet(A)* (Table 1), thereby conferring its multidrug-resistant phenotype. The backbone region of plasmid pRZ13-1 includes replication proteins (*repA*, *repHI2*), a plasmid partitioning system (ParAB), toxin–antitoxin systems (HipB-HigA, RelE/ParE), and a conjugative transfer region (Figure 2). The plasmid-encoded toxin–antitoxin module HipB-HigA not only promotes biofilm formation but also, under antibiotic stress, drives the generation of persister cells [47], thereby potentiating the durable multidrug resistance of RZ-13. The conjugative transfer region belongs to the Tra-type Type IV Secretion System (T4SS) (Figure 2), which facilitates the transfer of plasmid DNA and other molecules from the donor bacterium to the recipient bacterium [48]. Studies have demonstrated that the conjugative transfer of IncHI-type plasmids is temperature-sensitive, peaking at 22–30 °C and being markedly inhibited at 37 °C. Consistently, the transfer frequency of plasmid pRZ13-1 was 1.71 × 10^−6^ at 37 °C but rose to 2.70 × 10^−6^ at 24 °C, representing a 1.5-fold increase upon the 24 °C downward shift. Antimicrobial susceptibility testing demonstrated that the acquisition of pRZ13-1 conferred strain J53 resistance to sulfamethoxazole, chloramphenicol, gentamicin, doxycycline, kanamycin, ceftriaxone, ampicillin, and amoxicillin. This sustained, high-level conjugative transfer of pRZ13-1 across a broad temperature range enables the dissemination of its extensive resistance cargo among diverse bacterial hosts, posing a formidable threat to public health.

In addition to its extensive antibiotic-resistance arsenal, plasmid pRZ13-1 harbors the *terABCDEFZY1* operon, a complete tellurite-resistance cassette that confers the highest level of tellurite tolerance yet described. Within this cluster, *terC* and *terD* are the central determinants that reduce tellurite and therefore protect the cell; their individual over-expression arrests growth, but co-expression of either *terA* or *terZ* relieves this burden and restores normal physiology [49]. BLASTN analysis revealed that the entire ter module is 100% identical to segments carried on plasmids of a Canadian human *Salmonella Typhimurium* isolate (CP140756.1, Nov 2021) and a Chinese human *Klebsiella pneumoniae* strain (CP138804.1, 2021). Tellurium, a scarce metalloid of the chalcogen group, is enriched in mining waste and, before the penicillin era, tellurite was used as an antimicrobial agent; consequently, the increasing availability of microbial genome data now uncovers tellurite resistance as a far more common trait than previously appreciated [50,51,52].

**Figure 3:**
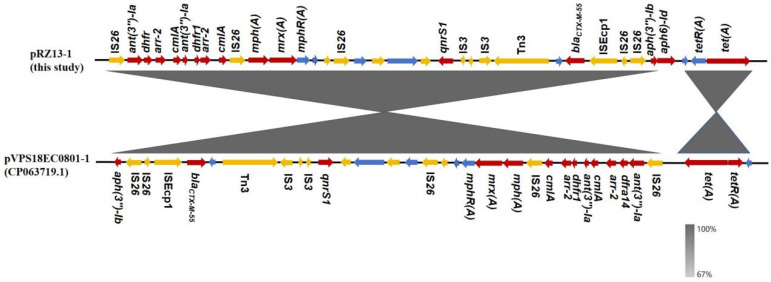
Partial nucleotide-sequence alignment of pRZ13-1 and pVPS18EC0801-1. ORFs are depicted as arrows indicating their position and transcriptional orientation; genes are color-coded: yellow for mobile elements, red for resistance determinants, and blue for hypothetical or other functionally annotated proteins.

Comparative sequence analysis revealed that plasmid pRZ13-1 shares a high degree of homology (99%) with plasmid pVPS18EC0801-1 (accession number: CP063719.1) derived from U.S. beef. The pVPS18EC0801-1 plasmid is a hybrid plasmid belonging to the IncN-IncHI2A-IncH12 incompatibility groups, and the host strains harboring both plasmids are of sequence type 345 (ST345) [53]. This suggests that they may originate from a common ancestor and have undergone clonal dissemination [54]. However, these two plasmids exhibit significant structural differences in their MDR regions (Figure 3). Compared with plasmid pVPS18EC0801-1, a region of approximately 29.4 kb in pRZ13-1 has undergone inversion. This inverted region contains multiple resistance genes, including *ant(3″)-Ia*, *dhfr*, *arr-2*, *cmlA*, *mph(A)*, *mrx(A)*, *qnrS1*, *tet(A)*, *bla_CTX-M-55_*, and *aph(3″)-Ib*. These resistance genes are flanked by multiple IS*26* elements, most of which are in the same transcriptional direction, with only one in the opposite direction. Meanwhile, two Tn3 family transposons with opposite orientations are also present in this region. This inversion may be mediated by inverted IS*26* elements and Tn3 transposases, and several IS*26* elements in the same direction were inserted after the inversion [55,56]. In addition, this region uniquely contains *terF*, *aph(6)-Id*, and multiple hypothetical proteins in sequence alignment. The *aph(6)-Id* gene is surrounded by IS*26* elements, indicating that *aph(6)-Id* may be mobilized via IS*26*-mediated transposition [57]. A recombinase family protein gene was also identified in this region, further suggesting that this region is a recombination hot spot for the evolution of resistance islands. Within this inverted region, a cluster of resistance genes flanked by IS*26* elements in the same direction was also observed, among which *ant(3″)-Ia*, *dhfr*, *arr-2*, and *cmlA* are arranged in a tandem manner. This hints at the possible occurrence of IS*26*-mediated tandem amplification or homologous recombination events. Such redundant gene arrangement may enhance the adaptability of the strain under the pressure of multiple antibiotics.

In addition, comparative analysis revealed that the MDR region of pRZ13-1 is highly similar to the corresponding regions in plasmid pEC71-IncHI2 (isolated from human-derived *E. coli* in China in 2020) and plasmid pCFSAN086837 (isolated from chicken-derived Salmonella in Vietnam in 2017). This further reflects the potential of such resistance structures for cross-host and cross-regional dissemination.

### 3.3. Characteristics and Transferability of the Multidrug-Resistance Plasmid pRZ13-3

**Figure 4:**
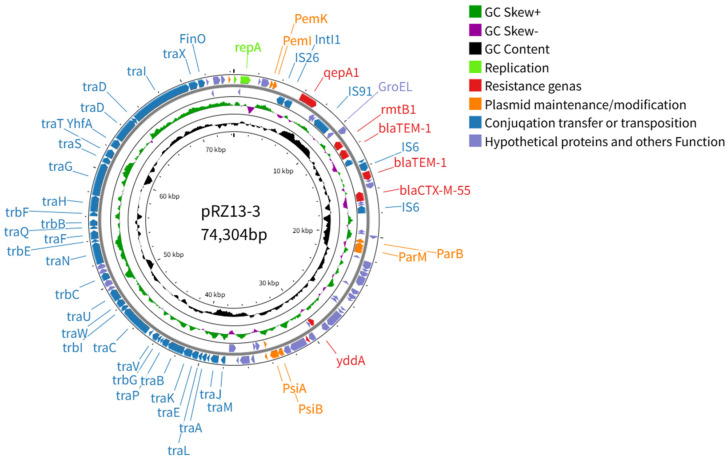
Circular map of plasmid pRZ13-3. The concentric rings (outermost to innermost) display predicted coding sequences, GC skew, GC content, and scale in kilobases; ORFs are color-coded by functional category. The complete map was generated with CG View (https://stothardresearch.ca/cgview/ (accessed on 24 April 2025)).

pRZ13-3 is an IncFII plasmid of 74,304 bp (53.00% G + C) that harbors 110 predicted ORFs, five of which encode resistance determinants: the β-lactamases *bla_CTX-M-55_* and *bla_TEM-1_* (two copies), the aminoglycoside-modifying enzyme *rmtB1* and the quinolone efflux gene *qepA1*. The plasmid backbone encodes the replication initiator RepA, three toxin–antitoxin systems (PemK/PemI, Sok/Hok and PsiB/PsiA) that ensure post-segregational killing [58], and the ParM/ParB plasmid-partitioning machinery that actively distributes copies to daughter cells [59] (Figure 4). Likewise, pRZ13-3 encodes a Tra-type T4SS that drives inter-strain transfer of the resistance plasmid. The conjugation assays yielded a frequency of 8.9 × 10^−2^, exceeding the transfer rates previously reported for IncFII plasmids of the same lineage [60]. Antimicrobial susceptibility testing demonstrated that the acquisition of pRZ13-3 conferred strain J53 resistance to gentamicin, doxycycline, ceftriaxone, ceftazidime, cefalexin, ampicillin, and amoxicillin.

**Figure 5:**
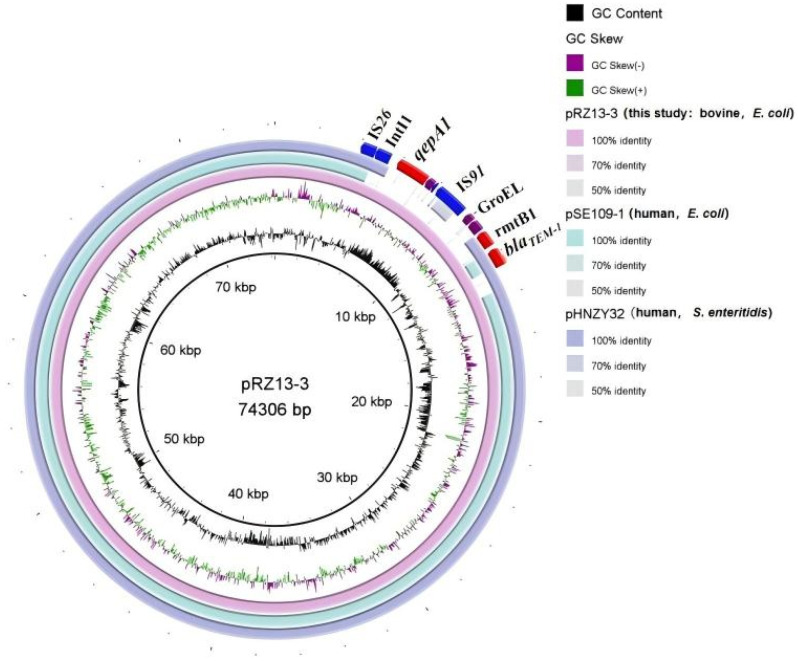
Genomic circular map comparing plasmid pRZ13-3 with two other plasmids. The innermost rings display GC skew (purple/green) and GC content (black). The remaining rings represent the genomic comparison of pRZ13-3, pSE109-1, and pHNZY32. The map was generated with BRIG.

BLAST analysis revealed that pRZ13-3 shares ≥99% nucleotide identity with numerous plasmids in GenBank (Table 2), three of which are of human origin, including two from *Salmonella enterica* serovar Enteritidis *(S. Enteritidis*). Whole-plasmid alignment with BRIG showed that pRZ13-3 is almost perfectly collinear with the Chinese human *E. coli* plasmid pHNZY32 and *S. Enteritidis* plasmid pSE109-1 across the T4SS-encoded conjugation region, toxin–antitoxin systems, replication machinery and plasmid-maintenance/modification modules (Figure 5). Divergence is restricted to a discrete island that carries *qepA1*, which confers fluoroquinolone resistance, the small-conductance mechanosensitive channel MscS, the Na^+^/H^+^ antiporter Cdu2, the molecular chaperone GroEL, a transposase-domain protein and the IS*91*-family transposase TPA. MscS and Cdu2 are predicted to enhance fitness under osmotic or acidic stress [61], whereas GroEL ensures stress-induced protein folding and has been implicated in bacterial adhesion, invasion and immune evasion [58]. The two transposases facilitate resistance-gene mobilization, and the flanking IS*91* elements indicate that the entire segment was acquired via IS*91*-mediated rolling-circle transposition [62]. This variable region is 100% identical to portions of the human-blood *E. coli* plasmid pEC-13-33-NDM-1 (China, 2013), the Vietnamese human Proteus mirabilis plasmid pMH13-009N_1 (2013; 99% identity), the bovine-diarrhea *E. coli* plasmid pHNXJB277-1 (Xinjiang, 2018) and the human-ascites *E. coli* plasmid pGYB02-2 (China, 2022), underscoring its repeated, inter-species and intercontinental dissemination.

## 4. Conclusions

This study provides a comprehensive genomic characterization of a multidrug-resistant and highly virulent extraintestinal pathogenic *Escherichia coli* (ExPEC) strain RZ-13 (ST345, O134:H21) isolated from diarrheic calves during a severe outbreak in China. Whole-genome sequencing revealed that the RZ-13 genome consists of a single chromosome and four plasmids, with two large conjugative plasmids, pRZ13-1 (IncHI2-IncHI2A, 265,777 bp) and pRZ13-3 (IncFII, 74,304 bp), serving as the primary reservoirs of antimicrobial resistance genes (ARGs). These plasmids collectively harbor over 25 resistance determinants conferring resistance to multiple clinically relevant antibiotic classes, including β-lactams, aminoglycosides, quinolones, tetracyclines, sulfonamides, and phenicols. Notably, the chromosome and plasmids carry four copies of the *bla_TEM-1_* gene, each embedded in distinct genetic contexts, suggesting that insertion sequence (IS)-mediated recombination and transposition events have contributed to the stable maintenance and potential amplification of β-lactam resistance.

A key finding is the structural complexity of the MDR region in pRZ13-1, which contains a 29.4 kb IS*26*/Tn*3*-mediated inverted region and a tandem array of resistance genes, indicating ongoing evolution through mobile genetic element activity. The presence of a complete tellurite resistance operon (*terABCDEFZY1*) further underscores the capacity of this plasmid to confer resistance to heavy metals, which may provide a co-selective advantage in agricultural environments. Moreover, pRZ13-1 exhibits temperature-sensitive conjugative transfer, with a 1.5-fold increase in frequency at 24 °C compared to 37 °C, highlighting its potential for dissemination in environmental and animal reservoirs where lower temperatures prevail.

Plasmid pRZ13-3, despite its smaller size, displays an exceptionally high conjugation frequency (8.9 × 10^−2^), far exceeding those previously reported for IncFII plasmids. It carries an IS*91*-mediated mobile island that integrates ARGs (*qepA1*, *rmtB*, *bla*_CTX-M-55_) with stress-adaptation genes (e.g., *mscS*, *cdu2*, *groEL*), suggesting co-selection of resistance and fitness traits. BLAST analysis revealed that pRZ13-3 shares ≥99% nucleotide identity with plasmids from human clinical isolates (e.g., *E. coli* and *Salmonella* from China) and animal sources (e.g., poultry in Vietnam), underscoring its broad host range and potential for inter-species and intercontinental dissemination.

The absence of classical enterotoxin or pneumonia-associated toxin genes in the RZ-13 genome, coupled with the presence of fimbrial, flagellar, and type VI secretion system effectors, points to a non-classical virulence mechanism whereby colonization, motility, and immune modulation collectively contribute to the observed diarrheic and respiratory symptoms. The lack of F5, F41, or F17 fimbrial antigens, which commonly associated with calf scours, suggests that other adhesins (e.g., type I fimbriae) may compensate in mediating intestinal adherence.

Collectively, these findings demonstrate that MDR plasmids in livestock-associated ExPEC can act as highly efficient vehicles for the horizontal transfer of resistance and virulence-associated genes, driven by complex MGE architectures and high-frequency conjugation. The close genetic relatedness of pRZ13-1 and pRZ13-3 to plasmids from human and animal isolates worldwide highlights the urgent need for integrated One Health surveillance to monitor and mitigate the spread of such high-risk clones across ecological niches. Future research should focus on elucidating the in vivo fitness costs and transmission dynamics of these plasmids, as well as exploring alternative control strategies to reduce the reliance on antimicrobials in livestock production.

## Figures and Tables

**Table 1:** Chromosome and plasmid profile of strain RZ-13.

Location	Size (bp)	GC%	No. of CDSs	Plasmid Type	Replicon Type	Virulence Factors	Resistance Genes
Chromosome	486,4281	51.43%	4759	–	–	–	*bla_TEM-1_*, *tet(A)*
pRZ13-1	265,777	46.80%	356	lncHI2-lncHI2A	*repB*, *repHI2*	*terC*	*aac(3)-IId*, *aph(6)-Id*, *aph(3″)-Ia*, *aph(3″)-Ib*, *bla_CTX-M-55_*, *bla_TEM-1_*, *ant(3″)-Ia*, *mph(A)*, *mef(B)*, *mphR*, *lnu(F)*, *floR*, *qnrS1*, *sul3*, *dfrA14*, *cmlA*, *arr-2*, *mrx(A)*, *tet(A)*, *terABCDEFZY1*
pRZ13-2	92,100	47.30%	132	ND	*repB*	*–*	*–*
pRZ13-3	74,304	53.00%	110	IncFII	*repA*	*traJ*, *traT*	*qepA1*, *rmtB*, *bla_TEM-1_*, *bla_CTX-M-55_*
pRZ13-4	61,618	42.38%	94	lncI2	*repA*	*–*	*–*

**Table 2:** Homology alignment results of plasmid pRZ13-3.

Plasmid	Size/bp	Query Coordinates/bp	Identity /%	Region	Isolation Source	Collection Date	Organism
EC9	4880,174	32,871~84,110	99	Hangzhou	Urine	September 2021	*E. coli*
pESBL-*bla_CTX-M-55_*	95,161	33,961~85,202	99	-	Chicken	-	*E. coli*
pHNZY32	145,804	85,267~136,504	99	Guangzhou	Human	-	*E. coli*
pSE109-1	90,315	29,116~80,347	99	-	Human	September 2013	*S. Enteritidis*
pSE104-1	106,652	45,447~96,684	99	Shanghai	Human	July 2013	*S. Enteritidis*

## Data Availability

This Whole Genome Shotgun project has been deposited at DDBJ/ENA/GenBank with the sequence data for Escherichia coli strain RZ-13 (BioProject: PRJNA1371668) under the accession number JBSTPK000000000.

## Abbreviations

ExPCEC, extraintestinal pathogenic *Escherichia coli*; MDR, multidrug resistance; *E. coli*, *Escherichia coli*; ARGs, antimicrobial resistance genes; MGEs, mobile genetic elements; HGT, horizontal gene transfer; WGS, whole genome sequencing; MLST, multilocus sequence typing; ISs, insertion sequences; *S. Enteritidis*, *Salmonella enterica* serovar Enteritidis.
